# Hexa­kis­(dimethyl sulfoxide-κ*O*)thallium(III) trinitrate

**DOI:** 10.1107/S1600536810022646

**Published:** 2010-06-18

**Authors:** Mohammad Ghadermazi, Faranak Manteghi

**Affiliations:** a Department of Chemistry, Faculty of Science, University of Kurdistan, Sanandaj, Iran; b Department of Chemistry, Iran University of Science and Technology, Tehran, Iran

## Abstract

The title compound, [Tl(C_2_H_6_OS)_6_](NO_3_)_3_, consists of six dimethyl sulfoxide (DMSO) mol­ecules coordinated to a Tl^III^ atom, which lies on a 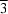 axis, and three nitrate anions (3. symmetry) to neutralize the charge. The coordination polyhedron around the Tl^III^ atom is octa­hedral, defined by six O atoms of the DMSO mol­ecules. In the crystal structure, C—H⋯O hydrogen bonds are observed. One of the nitrate groups exhibits half-occupation.

## Related literature

For general background to thallium(III) chemistry, see: Tóth & Gyõri (1994 ▶). For related structures, see: Aghabozorg, Ghadermazi *et al.* (2006 ▶); Aghabozorg, Ramezanipour *et al.* (2006 ▶); Ma *et al.* (2002 ▶); Notash *et al.* (2008 ▶).
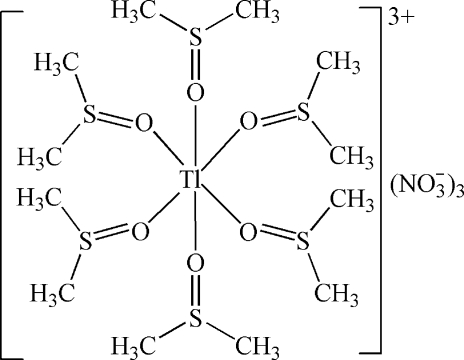


## Experimental

### 

#### Crystal data

[Tl(C_2_H_6_OS)_6_](NO_3_)_3_
*M* *_r_* = 859.17
Trigonal, 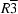

*a* = 11.7207 (9) Å
*c* = 19.209 (3) Å
*V* = 2285.3 (4) Å^3^
*Z* = 3
Mo *K*α radiation
μ = 5.78 mm^−1^
*T* = 100 K
0.23 × 0.12 × 0.04 mm

#### Data collection

Bruker APEXII CCD diffractometer
Absorption correction: multi-scan (*SADABS*; Sheldrick, 1996 ▶) *T*_min_ = 0.442, *T*_max_ = 0.786
9649 measured reflections
1480 independent reflections
1480 reflections with *I* > 2σ(*I*)
*R*_int_ = 0.036

#### Refinement

*R*[*F* ^2^ > 2σ(*F* ^2^)] = 0.022
*wR*(*F* ^2^) = 0.058
*S* = 0.99
1480 reflections
59 parameters
H-atom parameters constrained
Δρ_max_ = 1.97 e Å^−3^
Δρ_min_ = −0.76 e Å^−3^

### 

Data collection: *APEX2* (Bruker, 2007 ▶); cell refinement: *SAINT* (Bruker, 2007 ▶); data reduction: *SAINT*; program(s) used to solve structure: *SHELXTL* (Sheldrick, 2008 ▶); program(s) used to refine structure: *SHELXTL*; molecular graphics: *SHELXTL*; software used to prepare material for publication: *SHELXTL*.

## Supplementary Material

Crystal structure: contains datablocks I, global. DOI: 10.1107/S1600536810022646/hy2321sup1.cif

Structure factors: contains datablocks I. DOI: 10.1107/S1600536810022646/hy2321Isup2.hkl

Additional supplementary materials:  crystallographic information; 3D view; checkCIF report

## Figures and Tables

**Table 1:** Hydrogen-bond geometry (Å, °)

*D*—H⋯*A*	*D*—H	H⋯*A*	*D*⋯*A*	*D*—H⋯*A*
C1—H1*B*⋯O1	0.96	2.42	3.311 (4)	154
C1—H1*C*⋯O2^i^	0.96	2.54	3.448 (11)	158
C2—H2*A*⋯O1^ii^	0.96	2.55	3.380 (6)	145
C2—H2*B*⋯O2	0.96	1.99	2.915 (10)	161
C2—H2*C*⋯O1	0.96	2.55	3.423 (6)	152

Symmetry codes: (i) 
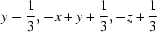
; (ii) 
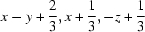
.

**Fig. 1.**
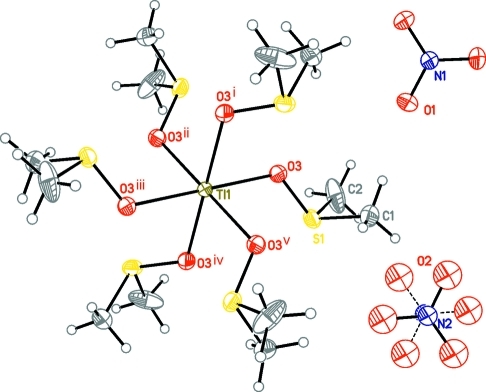
Molecular structure of the title compound. Displacement ellipsoids are shown at the 50% probability level. [Symmetry codes: (i) 1/3+x-y, -1/3+x, 2/3-z; (ii) 1-y, x-y, z; (iii) 4/3-x, 2/3-y, 2/3-z; (iv) 1-x+y, 1-x, z; (v) 1/3+y, 2/3-x+y, 2/3-z.]

**Fig. 2.**
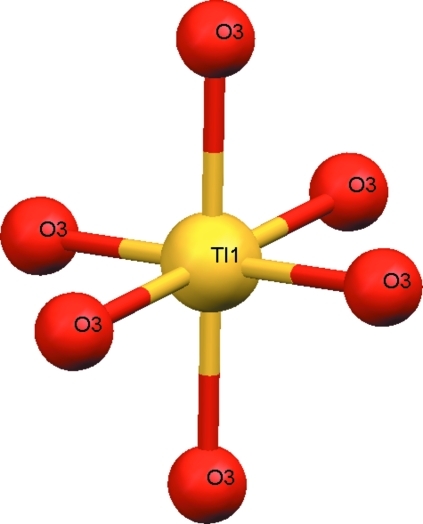
Coordination polyhedron around the metal centre.

## supplementary crystallographic information

### Comment

There are some reports on coordination of dimethyl sulfoxide (DMSO) as a neutral ligand to Tl(III), such as a triiodo complex [TlI_3_(DMSO)_2_] (Ma *et al.*, 2002) and [Tl(dm4bt)_2_(NO_3_)(DMSO)] (dm4bt = 2,2'-dimethyl-4,4'-bithiazole) (Notash *et al.*, 2008). Thallium(III) can be classified as a medium-soft metal ion in contrast to the other trivalent ions of group 13, aluminium(III), gallium(III) and indium(III), which are regarded as hard from their coordination properties (Tóth & Gyõri, 1994). The title compound has a coordination number of six around the metal (Figs. 1 and 2). However, the coordination numbers 4 to 9 are observed in different thallium(III) complexes (Aghabozorg, Ramezanipour *et al.*, 2006). Compared with [Tl(dm4bt)_2_(NO_3_)(DMSO)] and [TlI_3_(DMSO)_2_] mentioned above, in which the bond lengths of Tl(III) to O atoms of DMSO are 2.644 (7) and 2.468 (6) Å, the title compound has shorter Tl—O bonds [2.223 (2)–2.224 (2) Å]. This can be attributed to the less hindrance around the Tl centre. Also, compared with [Tl_2_(pydcH)_3_(pydc)(H_2_O)_2_] (pydcH_2_ = pyridine-2,6-dicarboxylic acid) (Aghabozorg, Ramezanipour *et al.*, 2006) and (pipzH_2_)[Tl_2_(pydc)_2_Cl_4_(H_2_O)_2_].4H_2_O (pipz = piperazine) (Aghabozorg, Ghadermazi *et al.*, 2006), whose Tl—O bond lengths vary in the range of 2.680 (4)–3.122 (4) and 2.436 (5)–2.508 (5) Å, respectively, again the Tl—O bond lengths in the title compound are obviously shorter. As shown in Table 1, only C—H···O hydrogen bonds can be seen in the lattice. The shortest C—H···O bond is C2—H2B···O2 with the best angle.

### Experimental

To a DMSO solution of Tl(NO_3_)_3_.3H_2_O (1 mmol, 443 mg) was added piperazinediium pyridine-2,6-dicarboxylate (3 mmol, 759 mg) prepared as literature (Aghabozorg, Ghadermazi *et al.*, 2006). The total volume of solution was 40 ml. The colourless crystals suitable for crystallography were obtained after six months.

### Refinement

H atoms on C atoms were positioned geometrically and refined as riding atoms, with C—H = 0.96 Å and *U*_iso_(H) = 1.5*U*_eq_(C). There is a high positive residual density of 1.97 e Å^-3^ near the Tl1 atom (distance 0.76 Å) due to considerable absorption effects which could not be completely corrected.

### Crystal data

**Table:**

[Tl(C_2_H_6_OS)_6_](NO_3_)_3_	*D*_x_ = 1.873 Mg m^−^^3^
*M**_r_* = 859.17	Mo *K*α radiation, λ = 0.71073 Å
Trigonal, *R*3	Cell parameters from 1197 reflections
Hall symbol: -R 3	θ = 2.3–34.3°
*a* = 11.7207 (9) Å	µ = 5.78 mm^−^^1^
*c* = 19.209 (3) Å	*T* = 100 K
*V* = 2285.3 (4) Å^3^	Plate, colourless
*Z* = 3	0.23 × 0.12 × 0.04 mm
*F*(000) = 1278	

### Data collection

**Table:**

Bruker APEXII CCD diffractometer	1480 independent reflections
Radiation source: fine-focus sealed tube	1480 reflections with *I* > 2σ(*I*)
graphite	*R*_int_ = 0.036
φ and ω scans	θ_max_ = 30.0°, θ_min_ = 2.3°
Absorption correction: multi-scan (*SADABS*; Sheldrick, 1996)	*h* = −16→16
*T*_min_ = 0.442, *T*_max_ = 0.786	*k* = −16→16
9649 measured reflections	*l* = −27→27

### Refinement

**Table:**

Refinement on *F*^2^	Primary atom site location: structure-invariant direct methods
Least-squares matrix: full	Secondary atom site location: difference Fourier map
*R*[*F*^2^ > 2σ(*F*^2^)] = 0.022	Hydrogen site location: inferred from neighbouring sites
*wR*(*F*^2^) = 0.058	H-atom parameters constrained
*S* = 0.99	*w* = 1/[σ^2^(*F*_o_^2^) + (0.040*P*)^2^ + 9.*P*] where *P* = (*F*_o_^2^ + 2*F*_c_^2^)/3
1480 reflections	(Δ/σ)_max_ < 0.001
59 parameters	Δρ_max_ = 1.97 e Å^−^^3^
0 restraints	Δρ_min_ = −0.76 e Å^−^^3^

### Fractional atomic coordinates and isotropic or equivalent isotropic displacement parameters (Å^2^)

**Table:**

	*x*	*y*	*z*	*U*_iso_*/*U*_eq_	Occ. (<1)
Tl1	0.6667	0.3333	0.3333	0.01735 (8)	
S1	0.47065 (6)	0.40706 (7)	0.24746 (4)	0.02613 (15)	
N1	0.0000	0.0000	0.2520 (2)	0.0268 (8)	
O3	0.48799 (19)	0.29141 (19)	0.27294 (11)	0.0235 (4)	
O1	0.1049 (2)	0.1084 (2)	0.25261 (16)	0.0383 (5)	
C1	0.3450 (3)	0.4021 (3)	0.30137 (19)	0.0333 (6)	
H1A	0.3792	0.4312	0.3474	0.050*	
H1B	0.2722	0.3136	0.3034	0.050*	
H1C	0.3158	0.4589	0.2824	0.050*	
C2	0.3806 (4)	0.3443 (5)	0.16924 (19)	0.0498 (11)	
H2A	0.4345	0.3328	0.1355	0.075*	
H2B	0.3554	0.4051	0.1515	0.075*	
H2C	0.3031	0.2610	0.1783	0.075*	
N2	0.3333	0.6667	0.1861 (4)	0.0280 (17)*	0.50
O2	0.3414 (8)	0.5668 (8)	0.1413 (4)	0.0609 (17)*	0.50

### Atomic displacement parameters (Å^2^)

**Table:**

	*U*^11^	*U*^22^	*U*^33^	*U*^12^	*U*^13^	*U*^23^
Tl1	0.01458 (8)	0.01458 (8)	0.02288 (12)	0.00729 (4)	0.000	0.000
S1	0.0176 (3)	0.0250 (3)	0.0332 (4)	0.0087 (2)	−0.0008 (2)	0.0090 (2)
N1	0.0221 (11)	0.0221 (11)	0.036 (2)	0.0110 (6)	0.000	0.000
O3	0.0209 (9)	0.0206 (8)	0.0287 (9)	0.0103 (7)	−0.0054 (7)	−0.0025 (7)
O1	0.0234 (10)	0.0229 (10)	0.0617 (16)	0.0063 (8)	−0.0014 (10)	−0.0006 (10)
C1	0.0278 (14)	0.0317 (15)	0.0436 (17)	0.0173 (12)	−0.0005 (12)	−0.0059 (12)
C2	0.0282 (16)	0.094 (3)	0.0273 (15)	0.0302 (19)	−0.0020 (12)	0.0109 (18)

### Geometric parameters (Å, °)

**Table:**

Tl1—O3^i^	2.2234 (19)	N1—O1^vii^	1.250 (2)
Tl1—O3^ii^	2.2235 (19)	C1—H1A	0.9600
Tl1—O3^iii^	2.2235 (19)	C1—H1B	0.9600
Tl1—O3^iv^	2.2235 (19)	C1—H1C	0.9600
Tl1—O3	2.2235 (19)	C2—H2A	0.9600
Tl1—O3^v^	2.2235 (19)	C2—H2B	0.9600
S1—O3	1.547 (2)	C2—H2C	0.9600
S1—C2	1.771 (4)	N2—O2^viii^	1.226 (8)
S1—C1	1.777 (3)	N2—O2^ix^	1.226 (8)
N1—O1	1.250 (2)	N2—O2^x^	1.226 (8)
N1—O1^vi^	1.250 (2)	O2—N2^x^	1.226 (8)
			
O3^i^—Tl1—O3^ii^	95.26 (7)	O1—N1—O1^vii^	119.993 (9)
O3^i^—Tl1—O3^iii^	180.0	O1^vi^—N1—O1^vii^	119.993 (9)
O3^ii^—Tl1—O3^iii^	84.74 (7)	S1—O3—Tl1	119.56 (11)
O3^i^—Tl1—O3^iv^	84.74 (7)	S1—C1—H1A	109.5
O3^ii^—Tl1—O3^iv^	180.0	S1—C1—H1B	109.5
O3^iii^—Tl1—O3^iv^	95.26 (7)	H1A—C1—H1B	109.5
O3^i^—Tl1—O3	84.74 (7)	S1—C1—H1C	109.5
O3^ii^—Tl1—O3	84.74 (7)	H1A—C1—H1C	109.5
O3^iii^—Tl1—O3	95.26 (7)	H1B—C1—H1C	109.5
O3^iv^—Tl1—O3	95.26 (7)	S1—C2—H2A	109.5
O3^i^—Tl1—O3^v^	95.26 (7)	S1—C2—H2B	109.5
O3^ii^—Tl1—O3^v^	95.26 (7)	H2A—C2—H2B	109.5
O3^iii^—Tl1—O3^v^	84.74 (7)	S1—C2—H2C	109.5
O3^iv^—Tl1—O3^v^	84.74 (7)	H2A—C2—H2C	109.5
O3—Tl1—O3^v^	180.0	H2B—C2—H2C	109.5
O3—S1—C2	102.52 (19)	O2^viii^—N2—O2^ix^	119.14 (17)
O3—S1—C1	104.88 (14)	O2^viii^—N2—O2^x^	119.14 (17)
C2—S1—C1	99.72 (17)	O2^ix^—N2—O2^x^	119.13 (17)
O1—N1—O1^vi^	119.993 (9)		
			
C2—S1—O3—Tl1	148.95 (15)	O2^x^—N2—O2—O2^viii^	112.8 (4)
C1—S1—O3—Tl1	−107.29 (16)	O2^xi^—N2—O2—O2^viii^	157.9 (4)
O3^i^—Tl1—O3—S1	46.66 (9)	O2^xii^—N2—O2—O2^viii^	67.8 (6)
O3^ii^—Tl1—O3—S1	142.45 (17)	N2^x^—N2—O2—O2^ix^	−112.8 (4)
O3^iii^—Tl1—O3—S1	−133.34 (9)	O2^viii^—N2—O2—O2^ix^	134.3 (9)
O3^iv^—Tl1—O3—S1	−37.55 (17)	O2^x^—N2—O2—O2^ix^	−112.8 (4)
N2^x^—N2—O2—O2^viii^	112.8 (4)	O2^xi^—N2—O2—O2^ix^	−67.8 (6)
O2^ix^—N2—O2—O2^viii^	−134.3 (9)	O2^xii^—N2—O2—O2^ix^	−157.9 (4)

Symmetry codes: (i) *y*+1/3, −*x*+*y*+2/3, −*z*+2/3; (ii) *x*−*y*+1/3, *x*−1/3, −*z*+2/3; (iii) −*y*+1, *x*−*y*, *z*; (iv) −*x*+*y*+1, −*x*+1, *z*; (v) −*x*+4/3, −*y*+2/3, −*z*+2/3; (vi) −*y*, *x*−*y*, *z*; (vii) −*x*+*y*, −*x*, *z*; (viii) *x*−*y*+2/3, *x*+1/3, −*z*+1/3; (ix) *y*−1/3, −*x*+*y*+1/3, −*z*+1/3; (x) −*x*+2/3, −*y*+4/3, −*z*+1/3; (xi) −*x*+*y*, −*x*+1, *z*; (xii) −*y*+1, *x*−*y*+1, *z*.

### Hydrogen-bond geometry (Å, °)

**Table:**

*D*—H···*A*	*D*—H	H···*A*	*D*···*A*	*D*—H···*A*
C1—H1B···O1	0.96	2.42	3.311 (4)	154
C1—H1C···O2^ix^	0.96	2.54	3.448 (11)	158
C2—H2A···O1^xiii^	0.96	2.55	3.380 (6)	145
C2—H2B···O2	0.96	1.99	2.915 (10)	161
C2—H2C···O1	0.96	2.55	3.423 (6)	152

Symmetry codes:  (ix) *y*−1/3, −*x*+*y*+1/3, −*z*+1/3; (xiii) *x*−*y*+2/3, *x*+1/3, −*z*+1/3.
